# MCH Pipeline Training Program: Connecting with Academia to Build Capacity Through Mentoring

**DOI:** 10.1007/s10995-022-03397-3

**Published:** 2022-03-24

**Authors:** Harolyn M. E. Belcher, Nikeea Copeland-Linder, Jacqueline Stone, Catrina R. Waters, Alice Kuo, Victoria Moerchen, Omonike Olaleye, Hamisu M. Salihu, Cheryl Vamos, Claudia Brown, Madhavi M. Reddy

**Affiliations:** 1 Kennedy Krieger Institute, Baltimore, MD USA; 2 Johns Hopkins University School of Medicine, Baltimore, MD USA; 3 Alabama State University, Montgomery, AL USA; 4 University of California-Los Angeles, Los Angeles, CA USA; 5 University of Wisconsin-Milwaukee, Milwaukee, WI USA; 6 Texas Southern University, Houston, TX USA; 7 Baylor College of Medicine, Houston, TX USA; 8 University of South Florida, Tampa, FL USA; 9 U.S. Department of Health and Human Services, Health Resources and Services Administration, Maternal and Child Health Bureau, Rockville, MD USA

**Keywords:** Culturally congruent mentorship, MCH workforce development, Underrepresented populations, Pipeline programs

## Abstract

**Introduction:**

Efforts to recruit and retain diverse Maternal and Child Health (MCH) professionals are of paramount public health significance. Culturally congruent mentorship strategies are key to supporting a successful transition from undergraduate to graduate studies.

**Methods:**

This mixed-method study evaluated a culturally congruent mentorship training used by one of the MCH Pipeline Training programs and described mentorship practices and lessons learned from the six MCH Pipeline programs. A retrospective pre-test post-test survey assessed mentorship competency skills following a mentoring workshop. All MCH Pipeline program leaders completed a questionnaire to elicit responses about mentoring training practices, mentor evaluation strategies, and lessons learned.

**Results:**

Maternal and Child Health Pipeline Training Programs supported 1890 undergraduate scholars at universities and institutions nationally. Scholars at six MCH Pipeline Programs participated in MCH education and mentored experiential leadership opportunities in clinical practice, research, and public health education. Qualitative program-level mentor survey themes indicated the importance of creating a reflective space and building mentorship teams. Mean mentor self-assessed improvement in mentor competencies was 14.4 points, 95% CI [10.5, 18.3], *p* < .001 following completion of a mentoring training workshop implemented by one of the MCH Pipeline programs.

**Discussion:**

The Health Resources and Services Administration’s Maternal and Child Health Bureau recognized the need to support the development of the next generation of diverse MCH leaders. Pipeline programs that included mentoring workshops and building culturally congruent mentorship teams are two strategies to increase and retain diverse scholars in graduate school and leaders in the public health workforce.

## Significance

A highly skilled and diverse maternal and child health (MCH) care workforce is vital to support the health of U.S. citizens. Mentoring is an effective tool that guides and promotes underrepresented scholars entering MCH and public health careers. Enhancing mentoring competencies through training workshops helps to build culturally congruent mentorship teams. Culturally congruent mentorship supports the success of scholars from diverse backgrounds.

## Introduction

Despite the Healthy People 2020 national priority to eliminate health disparities, women and children from racially/ethnically diverse and lower socioeconomic populations continue to have the highest rates of poor health, lower access to health care, and experience poorer health outcomes compared to the higher-income non-Hispanic white population (Agency for Healthcare Research & Quality, 2019; Centers for Disease Control & Prevention, 2013). The United States is becoming increasingly diverse. In 2015, of the 50.4 million children enrolled in public schools, kindergarten through twelfth grade, the majority were children of color: Hispanic/Latino (26%), Black (15%), Asian/Pacific Islander (5%), mixed race (4%), and American Indian/Alaska Native (1%) (deBrey et al., 2019). It is increasingly important that the healthcare workforce reflects the U.S. population.

The Maternal Child Health (MCH) workforce includes professionals in various fields dedicated to improving the health of mothers, children, and families. These fields include public health and related fields such as pediatrics, MCH nutrition, MCH social work, MCH nursing, pediatric dentistry, psychology, health education, pediatric occupational/physical therapy, and speech-language pathology. A 2017 study, Maternal and Child Health (MCH) Workforce Needs, estimated that 24% of the workforce planned to retire within 5 years and 28% planned to leave their agency within a year (Association of Maternal & Child Health Programs, 2017). This anticipated reduction of the MCH workforce and the burgeoning diversity of the U.S. population necessitates an immediate strategy to provide resources to build a diverse MCH workforce.

The Centers for Disease Control and Prevention Health Disparities and Inequalities Report noted inadequacies in health system infrastructure and access contributing to poor quality of care and outcomes among marginalized populations, including Black, Hispanic/Latino, and Native American/Alaska Native groups residing in areas with health professional shortages (Centers for Disease Control & Prevention, 2013). Patient-centeredness and effective provider-patient communication are enhanced by racial and ethnic concordance between patients and providers and providers’ cultural competency. Therefore, a diverse workforce is optimal (Agency for Healthcare Research & Quality, 2019). As the population of historically marginalized groups increases in the United States, academic institutions must address public health workforce shortages by recruiting students into MCH and public health from these racial and ethnic populations (Hollowell, 2015).

A lack of diversity in the MCH workforce may lead to cultural and linguistic barriers during patient–provider interactions, providers’ intentional or unintentional biases towards patients of different backgrounds, and providers’ clinical uncertainty when treating diverse populations. Studies found that marginalized racial and ethnic groups are more likely than non-Hispanic whites to report experiencing poorer quality patient–provider care. Most notably, individuals with limited English proficiency reported poor quality patient–provider care (HHS Action Plan to Reduce Racial and Ethnic Health Disparities, 2011). Conversely, workforce diversity is associated with greater patient satisfaction with healthcare and improved patient–provider communication. Diversifying the healthcare workforce is a crucial strategy to alleviate the severe health disparities in Black, Native American/Alaska Native, Hispanic/Latino, poor, disabled, and other marginalized populations in the United States due to systemic racism, other biases, and policies allocating resources.

### Health Resources and Services Administration’s (HRSA) Response

Goal 2 of HRSA FY 2019–2022 Strategic Plan fosters a health care workforce able to address current and emerging needs (Health Resources & Services Administration, 2019). Objective 2.1 under Goal 2 is to “Advance the competencies of the health workforce,” and Objective 2.2 is to “Improve the distribution and diversity of the health care workforce.” Specifically, HRSA-trained providers will address the social determinants of health and employ culturally competent communication, policies, and practices. HRSA is committed to increasing the diversity of the workforce and addressing shortages in the areas of greatest need (Health Resources & Services Administration, 2019).

MCHB’s Division of MCH Workforce Development supports trainees who show promise to become leaders in the MCH field through teaching, research, clinical practice, service, administration, and policymaking. The MCH Pipeline Training Programs address MCH Workforce development goals by providing early undergraduate scholars a “real world” opportunity to learn about MCH professions through research, clinical shadowing, education, and advocacy.

HRSA anticipated MCH workforce shortages and responded by developing the MCH Pipeline Training Program. The program’s purpose was “to promote the development of a culturally diverse and representative health care workforce by recruiting undergraduate training students from economically and educationally disadvantaged backgrounds (including racial and ethnic minorities) into MCH professions.” HRSA defined underrepresented racial and ethnic groups in the MCH professions as African American/Black, Hispanic/Latino, Asian, Hawaiian/Pacific Islander, and American Indian (Native American)/Alaska Natives. The MCH Pipeline programs were designed to recruit, educate, mentor, and provide professional experiences to prepare these new public health professionals for entry into MCH fields, such as pediatrics, obstetrics, nutrition, social work, nursing, pediatric dentistry, psychology, health education, occupational/physical therapy, and speech-language pathology*.* MCH Pipeline Training Programs supported 1890 scholars from six universities and institutions nationally. Scholars at each site participated in MCH education and mentored experiential leadership opportunities in clinical practice, research, community engagement, and advocacy.

A vital part of success for undergraduate scholars interested in MCH careers is effective mentorship that fosters trust and communication. An effective mentorship relationship will guide the scholar’s progress through the scholar’s academic and career paths and promote professional and leadership development. Mentors may serve as content experts and coaches. Mentors serve as sponsors by introducing scholars to opportunities and professional colleagues to foster the scholar’s success. Facilitating the scholar’s understanding of the cultural environment and expectations of the academic environment is a fundamental part of mentorship. Mentors should be responsive to the scholars’ culture and unique lived experiences. Culturally congruent mentorship is vital. Culturally congruent mentorship emphasizes building trust and communication while (1) promoting equity, diversity, and inclusion, (2) ensuring mutual understanding, (3) developing academic and research goals, (4) supporting independence, self-efficacy, and leadership, and (5) facilitating professional development and self-advocacy (Wyatt & Belcher, 2019).

The two-fold purpose of this mixed-method study was to: (1) evaluate a culturally congruent evidenced-based mentorship training workshop, Enhancing Mentoring, used by one of the MCH Pipeline Training programs, Maternal and Child Health-Leadership Education Advocacy Research Network (MCH-LEARN) Pipeline Program at Kennedy Krieger Institute and (2) describe mentorship practices and lessons learned from the six MCH Pipeline programs. It was hypothesized that Enhancing Mentoring training model and multi-modal mentoring strategies would improve mentoring skills. Sharing lessons learned across the geographically diverse MCH Pipeline Training programs may optimize mentoring practices for diverse undergraduate scholars interested in MCH careers.

## Methods

### Participants in Enhancing Mentoring Workshop

Forty-one senior clinical staff and faculty members participated in two Enhancing Mentoring workshops. The majority of the participants were female (*n* = 33, 80.5%) and white (*n* = 31, 75.6%). There were 17.1% (*n* = 7) Black and 7.3% (*n* = 3) Hispanic/Latino participants. Over half (*n* = 22, 53.7%) were faculty members (27.2% professors, 22.6% associate professors, 27.2% assistant professors, 9.0% instructors, and 14.0% postdoctoral fellows). The remaining participants (*n* = 19, 46.3%) were senior clinical staff.

### Mentorship Model for MCH-LEARN Pipeline Program

**Fig. 1:**
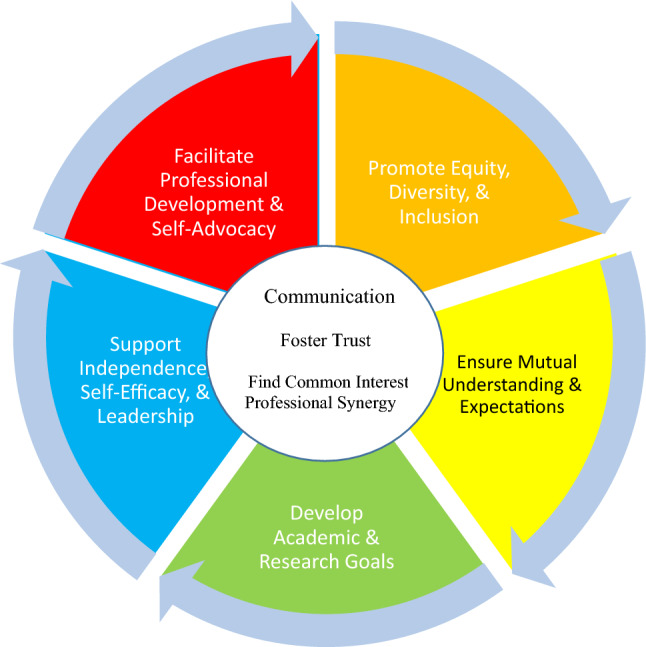
Mentors’ wheel: a culturally congruent foundation for mentoring scholars and faculty from underrepresented populations. © Belcher, Stone, Wyatt, 2019. ALL RIGHTS RESERVED

The Mentorship Model used for MCH-LEARN included an MCH research mentor and professional and academic mentor (PAM) for each undergraduate scholar. MCH research mentors and PAMs met weekly one-on-one with MCH-LEARN scholars. The MCH-LEARN program offered Enhancing Mentoring training to research mentors and PAMs. Enhancing Mentoring is an 8-h in-person workshop that focuses on strengthening and developing strong culturally congruent communication skills and trusting relationships. Enhancing Mentoring uses video vignettes of a diverse cohort of real-life scholars to facilitate discussions among mentors and develop methods to address important mentoring skills. The culturally congruent Enhancing Mentoring goals were summarized in the Introduction and illustrated in Fig. 1. The Enhancing Mentoring curriculum uses small group discussions, report-outs, role play, whole group discussions, and interactive learning. The first author’s Institutional Review Board approved the study.

### Assessments

#### Mentor Competency Assessment

The 26-item Mentor Competency Assessment (MCA) was used to measure self-report of mentor competency domains, including (a) Maintaining Effective Communication, (b) Aligning Expectations, (c) Assessing Understanding, (d) Addressing Diversity, (e) Fostering Independence, and (f) Promoting Professional Development (Fleming et al., 2013). The validation of the MCA used confirmatory factor analysis and maximum likelihood estimation to assess the fit of the 26 items with the competency domains. The data fit for the MCA for mentors’ responses was acceptable (chi-square = 663.20; df (284); *p* < .001, relative chi-square = 2.24, root mean square error of approximation = 0.069 90% confidence interval 0.062–0.076) (Fleming et al., 2013). A retrospective pre-test post-testing methodology measured the respondents’ reflections of their mentoring competency skills before and after participating in the Enhancing Mentoring workshop. Cronbach alpha for the 26-item MCA for the faculty cohort was 0.93. Cronbach alpha coefficients for mentor competency domains were as follows: communication: 0.78, aligning expectations: 0.91, assessing understanding: 0.57, fostering independence: 0.85, addressing diversity: 0.44, and promoting professional development: 0.72. MCA domains that aligned with cultural congruence characteristics of communication, trust, and equity, diversity, and inclusion were: (1) Maintaining Effective Communication, (2) Aligning Expectations, and (3) Addressing Diversity. The skills measured in the Maintaining Effective Communication domain include: listening, feedback, trust, styles, strategies, and coordinate (Fleming et al., 2013). The skills measured by the Aligning Expectations domain include: setting expectations, aligning expectations, considering personal and professional differences, setting goals, and developing strategies (Fleming et al., 2013). The skills measured by the Addressing Diversity domain include: considering personal biases and prejudice and working effectively with individuals from a different background (Fleming et al., 2013).

#### MCH Pipeline Program Questionnaire

MCH Pipeline program leaders constructed a questionnaire that collected processes for selecting mentors, mentor training practices, mentor evaluation strategies, and lessons learned. Themes were developed from open-ended responses.

### Statistical Analysis

Bivariate analyses were conducted using t-tests to analyze differences between mean pre-test and post-test scores on the MCA. Significance was set at an alpha of 0.05.

## Results

### Mentor Competency Assessment Post Enhancing Mentoring Workshop

Data from 21 mentor self-assessments were analyzed following the Enhancing Mentoring workshop. The mean baseline self-rating on MCA was 122.6 (*SD* = 15.1). The mean post-test following Enhancing Mentorship workshop score was 137.0 (*SD* = 10.4). The mean improvement on MCA self-rating was 14.4, (95% CI [10.5, 18.3], *p* < .001). Mean differences on all self-reported mentor competency domains demonstrated significant improvements (Table 1). The three mentor competency domains with the greatest average improvement ratings following the Enhancing Mentoring workshop were Maintaining Effective Communication, Aligning Expectations, and Addressing Diversity.

**Table 1:** Retrospective pre and post test of enhancing mentoring workshop evaluation (*N* = 41)

Mentor competency	Pre-test Mean (*SD*)	Post-test Mean (*SD*)	Mean difference (95% CI)
Maintaining effective communication (*n* = 21)	29.3 (3.7)	32.9 (3.2)	3.6 (2.6 to 4.6)***
Aligning expectations (*n* = 21)	23.5 (4.7)	27.4 (3.1)	3.9 (2.8 to5.0)***
Assessing understanding (*n* = 20)	13.5 (3.2)	14.1 (2.9)	0.6 (0.3 to 0 .9)***
Promoting independence (*n* = 20)	25.2 (4.0)	26.9 (3.3)	1.8 (0.8 to 2.7)**
Addressing diversity (*n* = 20)	9.4 (1.7)	11.5 (1.1)	2.2 (1.4 to 2.9)***
Promoting professional development (*n* = 21)	23.5 (4.5)	25.4 (3.6)	1.9 (1.0 to 2.8)***
Total (*n* = 17)	122.6 (15.1)	137.0 (10.4)	14.4 (10.5 to 18.3)***

*p < 0.05

**p < 0.01

***p < 0.001

### MCH Pipeline Mentor Training, Selection, and Evaluation

One program provided evidence-informed mentor training. One third of the programs indicated that they implemented systematic strategies to survey and select mentors related to their qualifications, past mentorship experience, and proposed mentorship experience. Mentor selection strategies for MCH Pipeline programs included: conducting site visits and program-specific training sessions. Half of the programs indicated that they used scholars’ evaluations of mentors’ courses or projects to inform mentor selection. The majority of programs indicated that their scholars completed evaluations of mentors and the mentoring experience. Additionally, programs reported assessing mentors on a variety of characteristics. Some of the most frequently evaluated mentor qualities include the mentor’s ability to promote professional development, knowledge of health disparities and equity practices, communication skills, foster leadership skills, and serve as a role model.

### MCH Pipeline Lessons Learned

MCH Pipeline directors shared lessons learned related to topics that ranged from the importance of supporting and engaging mentors to understanding the unique circumstances of underrepresented and first-generation college scholars. The following statements represent summaries of lessons learned from MCH Pipeline leadership responses.

- It is essential to give mentors guidelines to be clear about deliverables and expectations. Frequent check-ins with mentors are also helpful.
- It is helpful to have multiple mentors who may play distinct roles in the scholars’ experience.
- Formal mentor training may be beneficial for early career or graduate student mentors.
- It is helpful to recruit mentors and make mentor scholar matches early in the process.
- It is important to be aware of the unique psychosocial stressors faced by many underrepresented scholars or scholars from disadvantaged backgrounds. These stressors may include housing instability, the need to support family members while pursuing their education, financial stressors, and student feelings of guilt and conflict when not accessible to their families back home.
- Mental health and medical needs may impact scholars’ academic and social integration.
- Scholars may have gaps in their academic skills that require additional support.
- Mentees often struggle with issues related to imposter syndrome and stereotype threat. Mentors need resources and support structures that help them to process their own experiences related to imposter syndrome and stereotype threat.

## Discussion

This study examined mentor competencies following Enhancing Mentoring as an example of a mentorship training and evaluation model for MCH Pipeline programs. Mentors who participated in the Enhancing Mentoring workshop reported improvements in multiple mentorship domains. Mentor selection, training and evaluation strategies, and lessons learned from all MCH Pipeline programs were included. The MCH Pipeline leaders reported using various methods to evaluate mentors and the mentoring experience. A common strategy across programs was to gather scholar feedback on mentors, courses, and the mentoring experience.

Mentoring is one of the most important developmental relationships leading to academic and professional growth and success. Mentors support and facilitate the achievement of scholars’ goals. Notably, the MCA assessments’ highest mean improvement ratings were the Aligning Expectations, Maintaining Effective Communication, and Addressing Diversity domains. These three competency domains most closely align with skills characteristic of culturally congruent mentoring. Communication, active listening, and trust are central to culturally congruent mentoring. Aligning mentor and scholar expectations are fundamental to the mentoring experience. The Aligning Expectations domain measures multiple skills related to communication and respecting differences. Aligning expectations include developing strategies related to goal setting that respect different learning styles, skills, and aspirations. The Addressing Diversity domain requires self-reflection on the part of the mentor pertaining to acknowledging the mentor’s biases that may occur during the mentor–scholar relationship and understanding how to work with scholars who have different personal backgrounds from the mentor. Responses to these questions serve as the foundation for culturally congruent mentoring.

Providing evidence-informed mentor training such as Enhancing Mentoring may be one strategy to improve mentoring across multiple domains. The Enhancing Mentoring workshop uses actual student video vignettes to promote active discussion and cross-cultural communication. Improved expectations, ensuring understanding, promoting academic and career planning facilitates efficacy and leadership skills. Enhancing Mentoring uses a cultural and psychosocial lens to address potential scholar–mentor challenges and acknowledges the collective intellectual and experiential skills of academic mentors who participate in the training. MCH Pipeline program administrators identified several lessons learned, including the need for formal training of mentors, frequent check-ins between the scholar and mentors throughout the program, and mentors’ benefit helping scholars grow their network. By connecting scholars with other “mentor team members,” the scholar learns that throughout their career, they will need to seek out individuals with a variety of different skillsets to play different roles (e.g., counselor, technical expert) on their career journey. In addition, MCH Pipeline leaders identified the need for mentors to have resources to prepare them to address the needs of an increasingly diverse workforce. Formal training of mentors is needed to expand culturally congruent communication practices and strengthen evidence-based strategies and technical research skills, interpersonal skills, and cultural competence. Individual MCH Pipeline programs can create training programs to adapt and evaluate evidence-based strategies to address the unique aspects of underrepresented mentoring scholars.

MCH Pipeline programs must prepare the public health field to develop a more diverse public health workforce. Preparation of a diverse workforce requires professional development and support structures within training programs to address the needs of scholars from a variety of traditionally underrepresented backgrounds including, racial and ethnic populations, economically disadvantaged individuals, LGBTQ populations, and those with disabilities. To participate in culturally congruent mentoring, mentors need to prepare to engage in brave discussions about discrimination, bias, inclusivity, institutional barriers, imposter syndrome, and stereotype threats. For example, several MCH Pipeline program mentors noted that imposter syndrome was common among their mentees. Imposter syndrome, which was initially described by Chance and Imes (1978), refers to feelings of self-doubt in which a person does not feel deserving of their accomplishments or success and fears they will be exposed as a fraud. While imposter syndrome was initially used to describe high achieving women’s feelings (Chance & Imes, 1978), recent research indicates that imposter syndrome is more likely to occur among scholars from underrepresented backgrounds. Imposter syndrome has deleterious effects on mental health (Cokley et al., 2017). Mentors need to be aware of strategies to address imposter syndrome. Mentors may need “trusted” senior mentor-colleagues and support structures that allow mentors to process their own experiences with issues like imposter syndrome. This mentor-to-mentor support will promote authentic conversations with mentees, leading to effective strategies for coping with academic and personal challenges.

In conclusion, this paper evaluated a culturally congruent mentorship training used by one of the MCH Pipeline Training programs and described mentorship practices and lessons learned from the six national MCH Pipeline programs. As the scholar enters their graduate and work environment, mentoring and sponsorship become invaluable to guide successful career opportunities and advancement. The mentor–scholar relationship has dual benefits and responsibilities. Exploring, having curiosity, and understanding the scholar’s culture will enhance the trust and communication in the mentor–scholar interactions. Mentoring that results in culturally congruent relationships that are flexible and responsive to scholars’ needs and professional goals are essential. Culturally congruent mentoring efforts will contribute to the training, mentoring, and guiding of future MCH professionals, with the ultimate goal of eliminating health disparities through culturally congruent health equity practices and principles.
